# Valedictory Address to the Graduating Class of the 22d Annual Session of Rush Medical College, Jan. 25th, 1865

**Published:** 1865-05

**Authors:** E. S. Carr


### CHICAGO MEDICAL JOURNAL.

VOL. XXII.] MAY, 1865. [No. 5.

### ORIGINAL COMMUNICATIONS.

### VALEDICTORY ADDRESS TO THE GRADUATING CLASS OF THE TWENTY-SECOND ANNUAL SESSION OF RUSH MEDICAL COLLEGE, JANUARY 25th, 1865.

By Prof. E. S. CARR, M. D.

*Gentlemen of the Graduating Class:—*

It becomes my duty at this time to offer you the congratulations of your teachers in medicine, upon the successful termination of your pupilage, with a few parting words of advice and earnest well-wishing for your future career.

The word physician in its derivation from the Greek *phucis*, signifies a naturalist, one skilled in the operations of nature; and was only applied among the Greeks to their most eminent philosophers and learned men; such only being considered worthy to practice the art of healing. Among them was Hippocrates, justly considered the father of medicine, who says that whatever constitutes wisdom is to be found in medicine; meaning that the physician is, and of right ought to be, skilled in all human knowledge. In later times among the Romans the word "Doctor" came into use. The word literally means a teacher, as Gamaliel, a doctor of the law, meaning a teacher of, and one learned in the law. A doctor of physic or medicine, plainly implies one learned in physics or medicine—one who comprehends nature as *a whole*—whose studies and investigations render him so intimate with her laws that he can at pleasure apply his knowledge to the healing of the sick. Cicero tells us that men never resemble the gods so much as when giving life and health to their fellowmen. Such being their character and estimation in early times, we cease to wonder that Esculapius had his temple, and Hippocrates his statue, and that all along the stream of time are memorial statues for those who have fallen in the service of humanity.

It is not my purpose to consume the brief hour allotted to me in eulogizing the profession to which all of you have testified your devotion. You have all, I doubt not, entered it as earnest co-workers with the great and good of all ages, whose ambition has been the alleviation of all human misery, and the advancement of medical science. From each of you the world has a right to expect some golden grain to be added to the treasury of knowledge. Each of you may transmit the inheritance of past ages, brightened and purified in your personal experience and thought.

"Knowledge not realized is not knowledge for us, but knowledge's shadow." I wish to speak to you to-night of the relation which the Physician *should* sustain to the age in which he lives, and the community in which he is placed as the *minister and interpreter of nature*—of the development, present position and attainments, and possibilities of medical science, that you may gain a more adequate appreciation of the demands it makes upon its votaries.

In looking upon science simply as a basis of art, we are apt to depreciate the labors of those who have advanced it solely by their pursuit of abstract truth. And yet it is to these very men, who were observing, comparing, theorizing, two thousand years ago, that modern civilization is most indebted. Commerce to-day pays her grateful tribute to those old-time students of geometry, whose calculations made her path upon the waters.

Out of the dim shadows of antiquity, let us evoke to-night some of those spirits whose remote influences are still felt, who made the epochs in which they lived historical, and bid them tell us what, through the long years of their labor, they learned of the nature and causes of life.

Clad in the flowing garments of Greece, from the ruins of the temple of Cos, the figure of *Hippocrates* first responds tc to our call. "More than two thousand years ago," says the sage, "I laid the foundations of medical science; have I not written on imperishable papyrus that the human body is composed of three kinds of substances, solids, fluids and spirits,—did I not teach the multitude of my disciples that all diseases originate from an excess of the four *humours*, blood, phlegm, bile, and black bile? And for the cause of these mysteries, know you not that it is Nature, the superintending Intelligence? To look farther would awaken the displeasure of the gods."———"Yet, I looked," interrupts a nameless Alexandrian shade, "it was I who first took apart the House of Humanity, and in the body of a criminal found what Hippocrates had never dreamed of—the red and white machinery through which Life and Thought silently work—and bade Hierophilos my follower, count the pulsations which indicate their activity."

And now appears upon the stage a confused medley of actors. Dogmatics and Empirics, each claiming to be heralds of a great master.

"*I am Galen*," cries an advancing shade, "I united Dogmatism in reasoning, with Empiricism in practise, and sought the mystery of life in speculations of the highest order. Do not my books contain opinions which have, for twelve centuries, governed the philosophic world?"

Next appears that great crier, *Theophrastus Bombastus Paracelsus*, whose clamours first convoked men to hear the suggestions of experience. He bears in his hand a crucible, upon which "Eureka" is written in characters of fire; he opens his lecture thus:—

"I possess two sorts of knowledge;
One—vast, shadowy, hints of the
Unbounded aim I once pursued;
The other consists of many secrets, learned
While bent on nobler prize,—perhaps
A few first principles which may conduct to much.
These last I offer to my followers here."

"I shall be glad,
If all my labours, failing of aught else,
Suffice to make an inroad, and procure
A wider range for thought, nay, they do this,
For, whatsoe'er my notions of true knowledge
And a legitimate success may be,
I am not blind to my undoubted rank
When classed with others. I precede my age,
And whoso wills, is very free to mount
My labors as a platform."

"But if my spirit fail,
My once proud spirit forsake me at the last,
Hast Thou done well by me? So do not Thou!
Crush not my mind, dear God, though I be crushed!
Hold me before the frequence of Thy seraphs
And say, 'I crushed him lest he should disturb
My law. Men must not know their strength.
Behold, weak and alone, how near he raised himself.'"

Such was Paracelsus.

As the trees of the forest bend before the mighty wind, so do the spirits cower, as with solemn step and slow, the Interpreter of nature steps upon the scene. "My name and memory I did bequeath to men's charitable speeches, to foreign nations and the next ages," he saith. And again, "I perceive that medicine hath been more labored than advanced; there hath been much iteration, but little progression. Thou, oh Hippocrates, to whom Galen and Paracelsus betake themselves as to the shadow of an ass, thou wast a worshiper of idols, teaching that the secrets of nature are shut forever with the seal of God." "In thee, oh Galen, I do perceive a man that in his narrowness of mind forsook experience and became a vain pretender. Art thou not he who took away the infamy of ignorance and indolence in Physicians, by declaring so many diseases incurable, and defending thy incapacity by thy despair?"

"And thou, oh fanatical joiner of idols, with thy cohort of chemists beside thee, thou hast made man a pantomine! I can better endure Galen with his humours and elements, than Paracelsus adorning his dreams. Thy elders deserted experience, thou hast betrayed it, and polluted with thy vanity the truth thou mightest have unfolded."

I might continue this ideal representation of the teachers of antiquity a moment farther, bringing in the great cotemporary of Bacon, the immortal *Harvey*, and putting in his mouth the words of a living poet,

> "Life is life which generates,
> *Blood is blood which circulates,*
> And many-sided Life is One."

In so doing, I should only prove to you that the greatest importance of the past to us, is, in assisting us to determine the value of our present possessions.

Gœthe says that Bacon drew a wet sponge across the tablet of human knowledge. To realize the truth of this idea, place yourselves in thought in an early period of the Christian era, when the earth was regarded as reposing in the centre of the Universe, holding in her bosom, Man, the wonderful *Microcosm*, who with all Nature lived and was preserved through the influence of foreign planets, and we shall see how naturally, out of the physical and religious ideas of the time, arose *magic* and *astrology*. What were then considered the highest efforts of the magician tended to *separate* the Divine Element wherever it was found, sullied by opposing elements in visible nature, in order that the Divine and sustaining principle which lay concealed in every form, might act with freedom. Thus was Alchemy no chance and arbitrary thought, but a necessary element of the prevailing physics.

All physicists, and nearly all physicians searched for the philosopher's stone and the elixir of life, because they *believed* that in the world of gross matter the divine element, when purified, would transmute the same into gold and jewels; applied to the *microcosm* it had power to restore health and prolong life.

Many of the most distinguished alchemists were physicians and among them through the darkness of the middle ages, was hidden the learning and piety of the world. They interrogated nature with a prayer upon their lips; their superstition was the *faith* of the time. And despite the blindness and darkness of the middle ages, there shines out among the alchemists, a grandeur of character, a fixedness of purpose, and a purity of life we should do well to IMITATE.

Of Bacon, as of Plato, it may be said, "When he came, a man who could see two sides of a thing was born." Those pregnant apothegms, "Man is the servant and interpreter of Nature," "the limit of human power and knowledge is in the faculties alone," renovated the fountains of thought. Men heard with joy, such words as these, "God, the Creator of the world, has endowed our souls to contain that world; he has claimed our *faith* for Himself, but the world of *matter* He has submitted to our *senses*." In the inductive method instituted by this master mind, all things seemed contained, —the profits of experiment, the pleasure of details, the grouping of facts under principles, and those sublime generalizations which we call laws of nature.

Great as are the obligations of the scientist to Bacon, he was not alone. It was for Galileo, and Bruno, and Copernicus to enfranchise the human mind completely from the dominion of superstition,—to widen the universe for the exercise of its powers—to add to the lustre of great discoveries that of the crown of martyrdom.

For it was just at this period that *the Church* began the separation of religion from science, which was unknown in earlier times. *She* felt no security in her home upon an insignificant planet, whirling wildly round its fixed but distant centre; she tested the Copernican theory by the words of the Psalmist: "The sun is as a bridegroom coming out of his chamber, and rejoiceth like a strong man to run a race." Also, "the world is established that it can not be moved;" she tested it by the unaided senses, and found it wanting. Around the funeral pyre of Giordana Bruno, the battle of science and superstition began—nor did it cease after the Reformation had torn off the swaddling clothes in which the human spirit had been wrapped for ages.

In pursuing the evolutions of medicine since the time of Bacon, we observe that, while the objects of the profession have ever remained the same, and a patient search after the cause of life, the mysterious vital principle, continued, physicians abandoned hypotheses and confined themselves more and more to a diligent examination of phenomena. Harvey and Sydenham, and Jenner and Lænnec, produced by their discoveries the most wonderful change in practice. Yet the adoption of the new method was so gradual that even Sydenham considered the "heating of patients in small pox the readiest means of expelling their vicious humors." It is a remarkable fact, says Dr. Revere, that though no class of men have been so devoted to science as the members of our profession, into none of the sciences was the inductive system admitted with such reluctance. It first entered the domain of Anatomy, Physiology, and Surgery; the discovery of the lymphatics, the investigations of Charles Bell into the anatomy and effects of the nervous system being its first fruits. The discovery of Jenner, and the invention of the stethoscope were the results of pure induction. In the general practice of the profession progress has necessarily been much slower from the greater obscurity pervading all living processes, whether healthy or morbid.

Modern medicine dates from the commencement of the 17th century. No man became a convert to the opinions of Harvey till 1695, when Malpighi and Leuwenhock experimented with the microscope, and actually discovered the blood coursing through the arteries. * * * * Rich and varied as are the treasures which the faithful study of Nature have brought to our profession, there are still new continents to be discovereed. When we have exhausted our present domain of inquiries, Nature, like a kind mother, invites us to new fields of conquest.

> "Come wander with me, she saith,
> Into regions yet untrod;
> And read what is there to be read
> In the manuscript of God."

Only a small part of the secrets of the human constitution and the laws which govern it, are yet known to us. Life must be considered in its relations to surrounding media, nor can we thoroughly study man as an isolated object. Cuvier, Agassiz, and a host of lesser lights have wrought out the chain of his relationship to beast, bird, fish, mollusk, and moned, until all the successive steps between the highest and lowest forms seem unfolded to our view. And finally we must consider man as a religious, moral and intellectual, as well as physical being. And although the magnitude of these inquiries might almost deter us from entering upon them, all of the sciences are filling our hands with material for the work.

Medical education, and indeed all education, needs a larger infusion of positive science. Could we borrow time enough from the study of words, from barren metaphysical speculations, to learn what common sense has always dogmatically proclaimed to be of the first importance, the study of Humanity, the men of this generation might almost see the end of Charlatanry and Empiricism. The best interests of our profession demand, in addition to the regular practitioners, a trained class of special observers who may, by a rigid course of observation and experiment, use all available instrumentalities for the advancement of medical science.

But money must furnish the sinews of education as it does of war. Is it too chimerical to hope that, ere long the "solid men of Chicago" will lay broad and deep the foundations for Hospitals, museums, lecture rooms, and laboratories for instruction and investigation, which shall make the commercial metropolis of the West the medical metropolis of the Union?

Perhaps to some of you gentlemen, my remarks upon the claims and possibilities of our profession seem extravagant. You may say, to what end does the student burn the midnight lamp, consume weary days and nights in the wards of the hospital, inhale the fumes of laboratories, when the laurels he would win by honorable toil, the honors and emoluments of the profession, are bestowed by Esculapius upon his illegitimate children. I answer that this is only a partial truth, and it has this rational explanation. The majority of men, including many of the professedly educated, know so little of the foundations of medical science, that they never rightly estimate the value of the trained and the cultured physician. Were the masses of our people as well instructed in Hygiene as they are in Mathematics, they would cease to be victims of Quackery. They would be better able to realize what an amount of faithful preparation our profession requires, and only to that class of men who are aiding its development by scientific methods, and are governed by its principles in practice, would they entrust their lives.

Your intelligent neighbor knows very well that two and two makes four. He would not think of praying that the rusted corn in his granary be changed to healthy grain; but when the hand of sickness is laid upon his child, unwisely *fed* and *clothed* from its birth, his soul goes out in passionate appeals, and he grasps in his terror the first promise of relief. God forbid that I should speak with disrespect of those upward aspirations of the soul, which in sorrow and in extancy have ascended through all time to the "Father of Spirits,"

but I wish to illustrate the truth, which should be taught in the pulpit and the school, that *God works by laws*,—that it is our business to apply the powers and faculties he has given us to the understanding of these laws, which are but the expression of *His will ;* and that this, as well as obedience to them, is a moral duty.

The old Greeks were wiser than we, else had not Homer sung of Machaon, the pupil and son of Esculapias,

> "The spouse of Helen, dealing darts around,
> Had pierced *Machaon* with a deadly wound,
> In his right shoulder a broad shaft appeared
> And trembling Greece for her physician feared,
> To Nestor then Idomeneus began,
> Glory of Greece, old Nelius' valient son,
> Ascend thy chariot, haste with speed away,
> And great *Machaon* to the ships convey.
> A wise physician skilled our wounds to heal
> Is more than armies to the public weal."

Our country provides as yet no path of honor or preferment for those who serve her in camp and field with the weapons which science has forged for the preservation of life. At the call of patriotism, thousands of our noblest medical men left a lucrative professional business for the perils and discomforts of army life ; and yet, whatever their services, they are forced to remain satisfied without rank, preferment, or anything but the consciousness of faithful work. Many have laid down their lives in the noblest spirit of sacrifice. They died that their country and its defenders might live. But when they fall, "glory wakes no pains for them." Out of their desolated family circles, and the profession in which they have been honored members, none know their costly sacrifice. The popular eye is caught by the glitter of arms ; the popular ear is full of noisy triumphs ; but deeper down in the popular *heart* lies a true worship of the heroism of such unrewarded self-devotion, as many an army surgeon's career would furnish for the chronicler of this war. Let the warrior's grave have its laurels ; build high the sculptured marble over the statesman's breast, but lay the *faithful physician* under the daisies and the waiving grass, for the tears of the poor will water them, and his name be held in kind remembrance when brass and marble shall have crumbled into dust.

*Gentlemen Graduates* :—I have endeavored to present to you a standard of culture which contains all the elements of individual and professional greatness, and to which, I hope, you will aspire. May you each contribute largely to the sum of human knowledge, and aid in keeping up a close and vital union between medicine and general science. Be Doctors of Physic in the original and noble sense of the term, not mere dealers out of powders and pills—not as Luther called the Doctors of his time, "our Lord's cobblers,"—but skilful workmen in the laboratory of life. I need not tell you of the fearful responsibility which your difficult and laborious profession involves. It is not enough when at the bedside of the sick and suffering to say that we have done what we could. *Have we done all that could have been done?* Have we availed ourself of every fact and principle which our profession affords? If not, the pain, the agony, and perhaps the untimely death, stand justly chargeable to our account. Enter then upon your duties with an ardor that time cannot chill and a perseverance that shall overcome all obstacles. You have observed the close relation which exists between the development of the sciences and that of the material, moral and intellectual life of the race, and each of you will stand in the community where you are placed the minister and interpreter of nature. To no class of men does the popular mind turn with more entire confidence than to physicians. There are none who can so easily diffuse an interest in the attainment of scientific truth. Your daily walks and rides will give you opportunities for reading the outside of old earth's mysterious shield; in your reflective hours the mysteries of the inside will reveal itself. If you would not individually retrograde into the rear rank of the professional phalanx, you will be obliged to keep close upon the track of modern discovery and invention, to be students always. You will find in looking over the illustrious names of those who have done most in advancing medicine, that they were all zealous, busy practitioners of the healing art. Indeed

"No good, of worth sublime, does heaven permit,
To light on man as on the passing air,
The lamp of genius though by Nature lit,
If not protected, fed and pruned with care,
Soon dies or runs to waste with fitful glare."
"Has immortality of name been given
To them that idly worship hills and groves,
And burn sweet incense to the queen of heaven?
Did Newton learn from fancy, as it roves,
To measure worlds, and follow where each moves?
Or did Paul gain heaven's glory and its peace,
By musing o'er the bright and tranquil isle of Greece?"

I may seem to you to have presented science mainly with reference to its lower, material and most obvious uses, but, I firmly believe, beyond these it has rewards for you far more satisfying and permanent. The discoveries of the past are most valuable for their invigorating effect upon the mind of the present, stimulating it to apply the powers of nature to machinery and the mechanic arts, to all the arts of peace and war, they form the groundwork of present civilization; but it is when we realize that *every well conducted examination of a limited object discovers to us a part of the laws which govern the Infinite whole*, that we are led to its higher, more ennobling relations.

The literature of the ancients, embodying their first rude glances at Nature as breathed in the Iliad, will always charm and delight us; but the development of physical science, penetrating beneath the surface of things, showing us the connexion between the laws of nature and the laws of reason, teaching us that the whole creation is but the expression of an All Comprehensive idea, has opened to us a poetry and a literature as much above that of the ancients as knowledge is above ignorance, or truth above error. The true poet is a dreamer no longer.

"Earth outgrows the mythic fancies
Sung beside her in her youth.
And those debonaire romances
Sound but dull beside the truth.
Truth is fair, shall we forego it,
Shall we sigh, right, for a wrong?
God himself is the best poet,
And the real is his song.

Think what a glorious vision was his who first beheld through the telescope, planet after planet, world beyond world, with attendant satellites and moons! What new conception of the hidden glory of minute nature was his who revealed, by the aid of the microscope, a world in every atom! The eye within the eye, beholds the nature within the nature,—sees

"The One Spirit's plastic stress
Sweep through the dull, dense world, compelling there
All new successions to the forms they wear,
Torturing the unwilling dross that checks its flight,
To its own likeness, as each mass may bear,
And bursting in its beauty and its might
From trees and beasts and men into the heaven's light."

In conclusion, Gentlemen, let me assure you that, notwithstanding the vastness of the themes which a contemplation of nature presents, they have no tendency to diminish our interests in the daily affairs of life.

*He* who shared the counsels of the Father at creation's birth, the *Great Physician*, when among us healed the sick and comforted the afflicted. May you, each and all, imitate his example. See to it that the poor shall never need a friend, or our country in this hour of peril an unconditional supporter. May you lessen the burden of misery and ignorance in the world, and carry with you in all your labors, hearts touched by a *Divine* and universal *Love*.

Thus may you honor the profession which this day welcomes you to its ranks.

